# Congenital Anomalies of the Ossicular Chain: Surgical and Audiological Outcomes

**DOI:** 10.1177/00034894211025405

**Published:** 2021-06-11

**Authors:** Sara E. Henkemans, Adriana L. Smit, Robert J. Stokroos, Hans G.X.M. Thomeer

**Affiliations:** 1 Department of Otorhinolaryngology—Head and Neck Surgery, University Medical Centre Utrecht, Utrecht, the Netherlands; 2 Brain Centre Rudolf Magnus, University Medical Centre Utrecht, The Netherlands

**Keywords:** conductive hearing loss, minor ear anomalies, ossicular chain, congenital ear anomalies, exploratory tympanotomy, sensorineural hearing loss

## Abstract

**Objectives:**

In this study, we aim to analyze audiometric outcomes of middle ear surgery in patients with congenital middle ear anomalies.

**Methods:**

In this single center retrospective cohort study, audiological outcomes were extracted from patient files. Patients with a congenital middle ear anomaly treated surgically in a tertiary referral center between June 2015 and December 2020 were included. Pre- and postoperative short- and long-term audiometric data (at ≥3 and ≥10 months respectively) were compared to analyze hearing outcomes.

**Results:**

Eighteen ears (15 patients) were treated surgically with an exploratory tympanotomy. At short term follow up statistically significant improvements in air conduction thresholds and air-bone gaps were found. Hearing improved in 94.4% (17/18) of operated ears. Successful outcome, defined as an air-bone gap closure to within 20 dB after surgery, was reached in 44.4% (8/18). Serviceable hearing (air conduction ≤30 dB) was reached in 55.6% (10/18). Negative outcome (any significant deterioration in hearing) occurred in 1 patient: in this ear otitis media occurred during the postoperative course. At long term follow up, available for 50% of the cohort, hearing remained stable in 5 ears, improved in 1 ear and deteriorated in 3, all of which underwent revision surgery. Sensorineural hearing loss due to surgery, or other complications, were not encountered.

**Conclusion:**

middle ear surgery was found to be an effective treatment option to improve hearing in this cohort of patients with congenital middle ear anomalies. Surgical goals of obtained gain in air conduction thresholds and serviceable hearing levels were met by most patients without the occurrence of any iatrogenic sensorineural hearing loss.

## Introduction

A rare cause of congenital conductive hearing loss (HL) are congenital middle ear anomalies (CMEAs). They are defined as malformations of the auditory ossicles of any type.^1^ During the embryological development the auditory ossicles are formed from branchiogenic origin and malformations can occur at any stage of this development.^2^ Typically, conductive hearing losses of 30 to 50 dB are encountered in CMEA patients. CMEAs manifesting as isolated anomalies of the auditory ossicles are considered minor ear anomalies. When additional tympanic membrane or other external auditory canal anomalies (ie, atresia) are present, this deficit is described as a major anomaly.^1^ The incidence of CMEAs is around 0.28 per 100,000 persons,^3^ and CMEAs occur both uni- and bilaterally. CMEAs are predominantly sporadic, yet are also described to be part of a syndromal diagnosis in more than 25% of cases.^4^

Diagnosis of CMEAs can be significantly delayed, since conductive HL in children is common and often related to the high incidence of chronic otitis media with effusion (OME). Therefore, and regardless of a possible history of chronic OME in childhood, a CMEA needs to be considered in patients presenting with conductive or mixed HL later in life. A diagnostic delay is illustrated in many studies in which CMEAs were confirmed surgically in adult patients of 20 to 70 years of age.^5–17^ If ventilation tubes do not provide sufficient improvement of hearing in case of assumed OME, or if conductive hearing loss persists after resolution of OME, further audiological assessment and imaging of the middle ear, using high resolution computed tomography (HR-CT) are indicated for patients of all ages.^5,18^ When a CMEA is suspected, especially when both ears are affected, hearing improvement is usually sought to reach functional hearing levels. In these cases conventional hearing aids, bone conduction devices, and exploratory tympanotomy (ET) are options to be considered. Surgery may be postponed until children reach the age of 10 years to minimize the detrimental influence of postoperative otitis media on the reconstructed ossicular chain, and the risk of deaf ears due to subsequent labyrinthitis.^19^

CMEAs are most widely classified according to findings during ET using the Cremers and Teunissen classification (Table 1).^1^ In this grading system CMEAs are divided into 4 classes. According to the literature, class 1 anomalies are the most prevalent of all CMEAs.^12^ Though more recently, it is suggested that type 3 anomalies occur most often.^13-15,20-22^ In contrast to results in CMEA class 1 to 3 anomalies, surgery performed on class 4 anomalies has resulted in mixed long-term outcomes in several studies due to iatrogenic hearing loss and re-obliteration of the newly created window to the inner ear.^17,23,24^ Also, in several studies it is suggested that middle ear surgery in patients with specific syndromes can be less effective to restore hearing than in non-syndromal patients.^4,10,16^

**Table 1.** The Cremers and Teunissen^1^ Classification.

Class	Main anomaly	Subclasses
1	Isolated fixation of stapes footplate	
2	Fixation of stapes footplate, additional ossicular chain anomaly	a. Ossicular chain discontinuity
		b. Fixation in epitympanic recess
		c. Tympanic fixation
3	Ossicular chain anomaly, mobile stapes footplate	a. Ossicular chain discontinuity
		b. Fixation in epitympanic recess
		c. Tympanic fixation
4	Aplasia or dysplasia of oval or round windows	a. Aplasia
		I. Facial nerve abberancy
		II. Stapedial artery persistence
		b. Dysplasia
		I. Facial nerve abberancy
		II. Stapedial artery persistence

*Note*. Authors based this classification on surgical findings.

The decision to operate on CMEAs is made individually based on the estimated short and long-term benefit patients achieve after surgery, and is weighted against the possible risks during and after surgery (hearing deterioration, inner ear damage and chorda tympani or facial nerve lesion). This to attain maximal patient and parental informed consent in decision making before opting for surgery.

In this study we will assess hearing outcomes for a cohort of consecutively operated patients of varying ages with diverse anomalies of the middle ear classified as CMEAs. The aim of this study is to add to the existing literature regarding surgical outcomes in CMEA patients by presenting a unique cohort including all patients treated surgically during a 5-year period without excluding adults, syndromal patients, patients with otologic comorbidities, mixed hearing loss or a history of chronic OME as has often been the case in previously reported cohorts. These results will be compared to the current literature.

## Materials and Methods

### Study Design

This is a retrospective cohort study in which patient charts were reviewed to collect audiometric outcomes of patients with congenital middle ear anomalies surgically treated between June 2015 and December 2020. Patients were operated at the University Medical Center Utrecht The Netherlands, a tertiary referral center for middle ear disease. All surgical procedures were performed by experienced otological surgeons (DS, HT and RS).

Included patients were identified from the contributing surgeon’s personal databases. Inclusion criteria were: (1) patients with a pre- or peroperative CMEA diagnosis, (2) patients that received reconstructive middle ear surgery.

### Ethics

Ethical (non-WMO) approval for this study was granted by the local review board of the University Medical Center Utrecht, The Netherlands, local ethics number: 20-792. This study was conducted in accordance with the international ethical standard of the Helsinki declaration (2013).^25^

### Outcome Assessment

Surgical records were analyzed by one researcher (SH) to collect data about demographics, medical history of the patient (ie, previous surgeries and infectious events), type of surgical procedure performed, possible per- and postoperative complications and audiometric outcomes. The type of CMEAs, in all cases, were classified using the Cremers classification^1^ as displayed in Table 1.

Hearing outcomes were collected out of the audiometric evaluations within 4 months before surgery and at ≥3 months or longer (<10 months) after surgery to assess short-term postoperative outcomes. Hearing outcomes from the audiometric evaluation closest to 3 months postoperatively were used. To assess long term outcome, hearing outcomes measured at 10 months or longer postoperatively were collected. Hearing outcomes from the latest available audiometric evaluation >10 months postoperatively were used. If revision surgery was performed, the last available audiometry before revision surgery was used. Collection of audiometric data was done in accordance to the committee of hearing and equilibrium guidelines.^26^ All audiometric testing was performed in a sound treated room in the medical center’s facility by trained audiologists. Pre- and postoperative bone-conduction and air-conduction thresholds were measured at 0.5, 1, 2, and 4 kHz. Pure tone averages (PTAs) were calculated averaging these 4 values. Air-bone gaps (ABGs) were calculated using PTAs of BC and AC thresholds. When BC improved postoperatively (overclosure), the preoperative ABGs were corrected by using the postoperative BC. Successful hearing outcome was defined as closure of the ABG ≤ 20 dB.^27^ Serviceable hearing was defined as AC threshold ≤30 dB.^26^ A negative hearing outcome was defined by no change or any worsening in AC threshold PTAs or a worsening in BC PTAs ≥ 10 dB if the deterioration was a direct cause of the performed middle ear surgery. Hearing (AC) was considered stable if AC remained within 5 dB of the postoperative AC value used to calculate results. Individual short term audiometric results were visualized using Amsterdam Hearing Evaluation Plots (AHEPs).^27^

### Statistical Analyses

SPSS (IBM SPSS statistics 25.0.0.2) was used to perform all statistical analyses. Means, standard deviations and ranges were determined of pre- and postoperative audiometric results, that is, AC- and BC-threshold PTAs and ABGs. Using the Wilcoxon signed rank test preoperative audiometric results were compared to postoperative audiometric results for short and long term follow-up. A *P*-value of <0.05 was considered significant.

## Results

Fifteen patients (18 ears) underwent a total of 21 surgical procedures (18 procedures, 3 revision procedures) to restore middle ear function impaired by CMEAs. All patients had a history of hearing loss since early childhood. Other possible causes of hearing loss for example, ossicular fixation due to multiple episodes of otitis media or otosclerosis were excluded by combining anamnestic data with referrals and findings during exploratory tympanotomy.

Each ear and its specifications are displayed per patient in Table 2. Six patients were children and 9 patients were adults (8 and 10 ears respectively). Five patients had bilateral anomalies of which 3 patients underwent consecutive surgeries on either ear (Table 2). A syndromal diagnosis was encountered in 2 patients: branchio-oto-renal syndrome (BORs) in patient 10 (ear 11) and 22.q.11. deletion syndrome (22.q.11.ds) in patient 14 (ears 15 and 16). Both patients had bilateral hearing loss. As displayed in Table 2, 10 out of 18 ears had a medical history of chronic OME or tympanic membrane perforations. The anatomical anomalies encountered during ET and the performed procedures and placed prostheses are displayed in Table 3.

**Table 2.** Baseline Table of Patient Demographics.

Patient	Ear	Gender (M/F)	Side (AD/AS)	Bi- or unilateral HL (Bi/Un)	Age at surgery (years)	Relevant otological history	Anomaly Class	Syndromal diagnosis	Surgical revision (yes/no)
1	1	F	AD	Un	35	—	3	—	No
2	2	M	AS	Un	51	OM	2	—	No
3	3	M	AS	Un	62	—	3	—	Yes
4	4	M	AD	Un	19	—	3	—	No
5	5	F	AD	Un	25	—	2	—	No
6	6	M	AD	Un	56	OM,TMP	3	—	No
7	7	M	AD	Bi	18	OM	1	—	No
	8	M	AS	Bi	19	OM	1	—	No
8	9	F	AS	Un	15	—	3	—	Yes
9	10	F	AD	Bi	50	—	3	—	No
10	11	M	AS	Bi	27	OM	3	BORs	No
11	12	M	AD	Un	8	—	3	—	No
12	13	M	AS	Un	15	—	3	—	No
13	14	M	AD	Un	11	OM	3	—	No
14	15	F	AS	Bi	10	OM	3	22.Q.11.ds	Yes
	16	F	AD	Bi	11	OM	3	22.Q.11.ds	No
15	17	F	AS	Bi	11	OM	3	—	No
	18	F	AD	Bi	11	OM	3	—	No

Abbreviations: 22.q.11.ds, 22.q.11. deletion syndrome; AD, auris dexter; AS, auris sinister; BORs, branchio-oto-renal syndrome; F, Female; M, male; OM, otitis media; TMP, tympanic membrane perforation.

**Table 3.** Surgical Findings, Procedures Performed and Placed Prostheses.

Ears	Operative findings	Procedure/prosthesis	Ears	Operative findings	Procedure/prosthesis
1	Ossification of the stapedial tendon	OCM	10	Only membranous connection between incus and stapes	OCR: Otomimix Olympus^®^, Hamburg
2	Epitympanic fixation incus Stapes footplate fixation	STPdo + OCR: Fluoroplastic Loop Piston, Medtronic^®^	11	Fragile incus No adequate connection incus and stapes	OCR: Tutoplast incus ATGMED - AT TECHNOLOGIES GmbH^®^
3	Absent long process of incus	OCR: PORP clip Kurz Medical^®^, Dresden	12	Atrophy of long process incus Absent anterior crus stapes Only membranous connection between incus and stapes	OCR: TORP Kurz Medical^®^, Dresden
4	Fixated incudomalleolar joint Absent stapes suprastructure No connection between stapes and incus	OCR: TORP Kurz Medical^®^, Dresden	13	Fixated incudomalleolar joint Fragile connection between long crus incus and stapes	OCR: PORP clip Kurz Medical^®^, Dresden
5	Fixation of the stapedial suprastructure to the osseous facial nerve covering Stapes footplate fixation	STPdo + OCR: Fluoroplastic Loop Piston, Medtronic^®^	14	Fixation of the mallear manubrium, epitympanic fixation of the mallear head	OCM
6	Absent incus Absent stapes suprastructure	OCR: TORP Kurz Medical^®^, Dresden	15	Anterior fixation of the mallear manubrium	OCM
7	Monopodal stapes suprastructure, consolidated with the stapes footplate Partially ossified stapedial tendon Stapes footplate fixation	STPde + OCR: Fluoroplastic Loop Piston, Medtronic^®^	16	Fixated malleus Fragile long crus incus, absent lenticular process Fragile connection between incus and stapes	OCR: PORP clip Kurz Medical^®^, Dresden
8	Monopodal stapes suprastructure, consolidated with the stapes footplate Partially ossified stapedial tendon Stapes footplate fixation	STPde + OCR: Fluoroplastic Loop Piston, Medtronic^®^	17	Fixated incudomalleolar joint Short long process incus, fragile connection with stapes	OCR: PORP clip Kurz Medical^®^, Dresden
9	Fixation of the anterior mallear ligament	OCM	18	Fixated incudomalleolar joint Short long process incus, fragile connection with stapes	OCR: PORP clip Kurz Medical^®^, Dresden

*Note*. The anatomy of congenital middle ear anomalies encountered during TE, subsequent procedures performed, and the placed allogenic material in OCR surgeries in all operated ears. A prosthesis was placed in all OCR surgeries.

Ears 7 and 8 underwent STPde surgeries as the consolidated stapes suprastructure/footplate made regular stapedotomy surgery impossible.

Abbreviations: OCM, ossicular chain mobilization; OCR, ossicular chain reconstruction; PORP, partial ossicular replacement prosthesis; STPde, stapedectomy; STPdo, stapedotomy; TORP, total ossicular replacement prosthesis.

### Surgical Methods per CMEA Class

**Figure 1.**
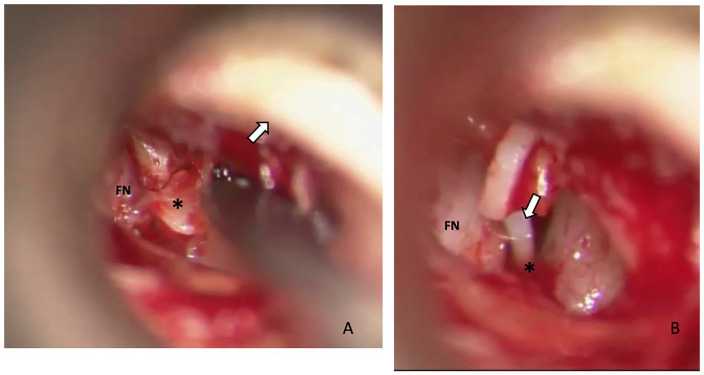
Stapedectomy procedure, Endaural view Right Ear in patient 7 (class 1 anomaly). (A) Luxation and removal of stapes footplate with monopodal stapes head (*). Facial nerve (FN) and the tympanomeatal flap (arrow) are indicated. (B) Piston (All Teflon, Causse Loop) (arrow) is placed around long process of the incus and positioned in the vestibulum (*). Facial nerve (FN) is indicated. Pre- and postoperative AC: 35 and 22.5 dB, respectively.

All surgical procedures were performed under general anesthesia, using a trans-canal endaural (16 procedures) or retro-aural approach (5 procedures) to reach the middle ear. The used technique for ossicular chain reconstruction differed depending on the found anatomical anomalies of the ossicular chain and consequential classifications. For class 1 anomalies a stapedectomy procedure was performed (figure 1): the stapes was removed and replaced by a Teflon Causse piston (Fluoroplastic loop piston, Medtronic®). For class 3 anomalies, either removal of fixed bone, or total or partial replacement of the ossicular chain by a prosthesis, ossicular chain reconstruction (TORP or PORP respectively, Kurz Medical^®^, Dresden), was performed. For class 2 anomalies a combination of a stapedotomy procedure and the reconstructions conducted in class 3 anomalies was performed. During stapedotomy the stapes suprastructure was removed, the footplate was perforated (using a 1W KTP laser) and a Teflon Causse piston (Fluoroplastic loop piston, Medtronic®) was placed.

### Short Term Results

**Figure 2.**
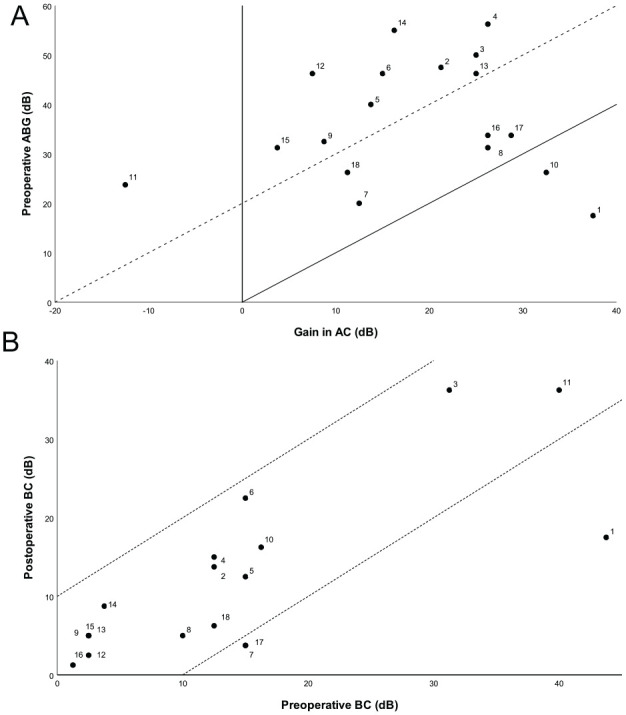
Amsterdam hearing plots. (A) AHEP A: Audiological outcome after middle ear surgery per operated ear (mean FU 4.0 [1.4-8.1] months). Gain in AC plotted against preoperative ABG (not corrected for overclosure) in dB. The solid diagonal line marks complete closure of the ABG. The area in between the dotted and solid diagonal lines marks ABG closure to ≤20 dB. The area under the solid diagonal line marks a gain in AC greater than expected based on the preoperative ABG due to overclosure. (B) AHEP B: Audiological outcome after middle ear surgery per operated ear (mean FU 4.0 [1.4-8.1] months). Preoperative BC is plotted against postoperative BC. The area in between the dotted diagonal lines marks change in BC < 10 dB. The area under the lower diagonal line indicates significant BC improvement (>10 dB). The area above the upper diagonal line indicates SNHL (>10 dB). *Note*. Ears 9, 13, and 15 and ears 7 and 17 share the same spot on this AHEP.

Short-term postoperative hearing outcomes were available for all 18 ears and are displayed in Table 4 and visualized per operated ear in AHEPs in Figure 2. The mean duration of short-term follow up was 4.0 months (range 1.4-8.1 months). Audiometric results at FU ≥ 3 months were available for eleven ears. In this cohort of patients the mean gain in AC (18.1 dB ± 12.0) and closure of the ABG (19.7 dB ± 12.2) were found to be significant (*P* < 0.001), comparing the preoperative and short term postoperative measurements. In 17 out of 18 ears (94.4%) improvement of AC was obtained comparing preoperative measurements to short term postoperative measurements. Successful outcome (ABG ≤ 20 dB) was reached in 8 out of 18 (44.4%) ears (specifically, in 3 out of 8 children (37.5%) and 5 out of 10 adults (50.0%). Serviceable hearing levels (AC ≤ 30 dB) were reached in 10 out of 18 ears (55.6%), in 75% (6/8) of children and in 40% (4/10) of adults. No postoperative decline in bone conduction hearing levels (sensorineural hearing loss) were seen in this study. Negative outcome was seen in one (5.6%) out of 18 ears. In this patient (ear 11, class 3 CMEA, syndromal hearing loss by BORs) a postoperative acute otitis media occurred 3 weeks after ossicular chain reconstruction (OCR) surgery. At hearing evaluation 6 weeks after surgery an AC PTA of 76.3 dB, BC of 36.3 dB and ABG of 40.0 dB were found compared to preoperative PTAs AC 63.8 dB, BC 40.0 dB and ABG23.8 dB. These hearing levels remained stable during the 5 years of FU after surgery. It was patient’s and doctor’s shared decision that revision surgery should not be performed as the low probability of relevant hearing improvement did not outweigh the risk of a postoperative deaf ear.

**Table 4.** Mean Pre- and Postoperative Audiometric Values at Short Term Follow Up.

	Class (n)	AC (dB)	BC (dB)	ABG (dB)
Preoperative	Class 1 (2)	38.1 (35.0-41.3) ± 4.4	12.5 (10.0-15.0) ± 3.5	33.8 (31.3-36.3) ± 3.5
	Class 2 (2)	57.5 (55.0-60.0) ± 3.5	13.8 (12.5-15.0) ± 1.8	45.0 (42.5-47.5) ± 3.5
	Class 3 (14)	51.9 (33.8-81.3) ± 14.3	14.4 (1.25-43.8) ± 14.3	40.9 (26.3-56.3) ± 10.0
	Total (18)	51.0 (33.8-81.3) ± 13.6	14.1 (1.25-43.8) ± 12.6	40.6 (26.3-56.3) ± 9.3
Postoperative	Class 1 (2)	18.8 (15.0-22.5) ± 5.3	4.4 (3.8-5.0) ± 0.9	14.4(10.0-18.8) ± 6.2
	Class2 (2)	40.0 (38.8-41.3) ± 1.8	13.1 (12.5-13.8) ± 0.9	26.9 (25.0-28,8) ± 2.7
	Class 3 (14)	33.9 (8.8-76.3) ± 18.3	12.9 (1.3-36.3) ± 11.8	21.0 (-6.3)-40.0) ± 12.6
	Total (18)	32.9* (8.8-76.3) ± 17.0	12.0 (1.3-36.3) ± 10.7	20.9* (-6.3)-40.0) ± 11.6

*Note*. Mean audiometric values (range) ± SD (mean FU 4.0 [1.4-8.1] months).

* Changes in AC and ABGs were statistically significant.

### Long Term Results

Long term (FU ≥ 10 months) postoperative hearing outcome was available for 9 out of 18 ears (50.0%). The mean duration of long-term follow up was 23.6 months (10.8-58.9 months). Mean postoperative audiometric values were: BC 13.8 dB, AC 44.3 dB, and ABG 30.6 dB (compared to BC 16.3 dB, AC 50.8 dB, and ABG 38.2 dB preoperatively and BC 14.7 dB, AC 38.2 dB, and ABG 23.5 dB short term follow-up for these ears). Postoperative hearing outcomes remained stable in 5 out of 8 cases (ears 2, 6, 7, 11, and 18) and improved in 1 case (ear 17). Hearing declined in the other 3 cases (ears 3, 9 and 15), which were all class 3 CMEAs, all of which underwent revision surgery.

### Revision Surgery

Postoperative hearing in ear 3, after PORP (Kurz Medical®, titanium prosthesis) placement to correct a missing long process of the incus, improved from BC 31.3 dB, AC 81.3 dB, and ABG 50.0 dB preoperatively to BC 36.3 dB, AC 56.3 dB, and ABG 20.0 dB postoperatively. Recurrence of conductive HL occurred (BC 33.8 dB, AC 78.8 dB, ABG 45.0 dB) 19 months after surgery. Preoperative HR-CT before revision surgery demonstrated a correct position of the used ossicular reconstruction prosthesis. During revision ET the previously placed PORP appeared too short (length 2.0 mm) and a new, longer PORP (both Kurz Medical^®^, titanium prosthesis) (length 2.5 mm) was placed. This resulted in a subjective improved hearing directly postoperatively, however 6 days after surgery hearing loss without vertigo or instability occurred in this ear (BC 65.0 dB, AC 91.3 dB) which was interpreted as sudden deafness and treated accordingly with 7 days of prednisone. HR-CT and MRI did not show abnormalities of the middle ear and position of the placed prosthesis. Hearing remained stable in the 2 months follow up after this event (BC 66.3 dB and AC 88.8 dB) and no further explanation for the HL was found.

After initial surgery in ear 9, in which the fixation of the anterior mallear ligament was removed, hearing improved according to the patient and then gradually declined in the 8-week period following with audiometrical outcomes of BC 8.8 dB, AC 27.5 dB, and ABG 18.8 dB compared to BC 2.5 dB, AC 35 dB, and ABG 32.5 dB preoperatively. Revision ET revealed recurrent fixation of the anterior mallear ligament. The incus and fixated malleus head were removed, and a PORP (Kurz Medical^®^, titanium prosthesis) was positioned on the mobile stapes suprastructure. Hearing and audiometric PTAs improved (BC 2.5 dB, AC 12.5 dB, ABG 10 dB) and remained stable during follow up (5 months).

Surgery in ear 15 (hearing loss by 22.q.11.ds), in which the anterior fixation of the mallear manubrium was removed, resulted in little hearing improvement (postoperative BC 5.0 dB, AC 30.0 dB, and ABG 25.0 dB compared to BC 2.5 dB, AC 33.8 dB, and ABG 31.3 dB preoperatively). Audiometry 1 year post surgery showed deterioration in hearing levels (BC 5.0 dB, AC 61.3 dB, ABG 56.3 dB). Revision surgery was performed in which the long leg of the incus was removed and a PORP (Kurz Medical^®^, titanium prosthesis) was placed resulting in improved hearing (BC −1.3 dB, AC 26.3 dB, ABG 27.5 dB).

We encountered no surgery related complications in this cohort such as direct postoperative sensorineural hearing loss or deafness, iatrogenic damage to the facial nerve or persistent postoperative vertigo/dizziness.

## Discussion

In this single center, retrospective study the hearing outcomes of 18 ears after surgery for congenital anomalies of the middle ear were assessed. Surgical intervention for CMEAs improved AC thresholds and ABGs significantly. 50.0% (9/18) of ears had ABGs ≤ 20 dB after surgery (and revision surgery) compared to zero out of 18 before surgery. The results of this study were compared to previously published patient series that reported hearing outcomes in larger patient cohorts (n ≥ 10) and followed the AAO-HNS committee guidelines for reporting hearing outcomes.

### Classification and Surgical Outcome

In the literature good results of surgery on class 1 ears have been reported with mean postoperative ABGs of 11 to 14 dB and success rates of 74%,^6,28,29^ which is comparable to our experience (mean gain in AC 19.4 dB, postoperative AC 18.8 dB, ABG 14.4 dB and success rate 100%). Iatrogenic sensorineural hearing loss (SNHL), although rare, is reported to occur most frequently in CMEA class 1 cases (0% to 4%)^6,28,29^ compared to the other classes of CMEAs. This can be explained, considering the increased risk of an infectious event after opening the inner ear during stapedectomy procedures. No surgery related SNHL occurred in our cohort.

Literature in which hearing outcome on class 2 patients is reported is limited. In the few reports that published outcome in class 2 patients an AC gain of 18 to 20 dB has been found, with higher postoperative ABGs (13-20 dB) and lower success rates (67% to 70%) than in class 1 and class 3 patients.^10-13,29^ The presented cohort included 2 class 2 ears in which a gain in AC was achieved of 17.5 dB and success rate of 0% (mean postoperative AC 40.0 dB and ABG 26.9 dB). Ear 2 had an otologic history of multiple episodes of otitis which is likely to have limited final hearing outcome. During surgery on this ear atelectasis of the tympanic membrane and many adhesions of the middle ear were found.

Most ears in this study (14/18) were classified as class 3. Results on class 3 ears presented in the literature are summarized in Table 5 and compared to the short term audiometric outcome of our study.^16,20,22,30,31^ The mean postoperative ABG and gain in AC achieved in our cohort are rather similar to published case series, but success rates were lower (44.4% in our cohort compared to a range from 56.1% to 75.0% in the literature). These lower success rates might be explained by the significant amount of ears (7/14) with an otologic history of chronic OME. Additionally, 3 of these ears (ears 6, 11, 14) presented with high AC PTA’s and large ABGs which decreases the probability of closing the ABG to within 20 dB. Furthermore, the class 3 group included all 3 syndromic ears of which 2 (ears 11 and 15) did not acquire a postoperative ABG within 20 dB.

**Table 5.** Class 3 Short Term Outcomes Compared to the Literature.

Class 3	Current study (2020)	Sakamoto et al.^30^	Thomeer et al.^16,17^	Philippon et al.^22^	Vincent et al.^29,31^	Quesnel et al.^20^
No ears	14	41	23	18	16	19
Follow up (m [range])	4 (1-9)	12	12	6	67 (6-169)	35
Preoperative						
Mean AC (dB)	52	60	47	50	45	50
Mean BC (dB)	14	21	9	9	14	13
Mean ABG (dB)	41	39	38	41	31	37
Postoperative						
Mean AC (dB)	34	41	30	30	23	29
Mean BC (dB)	13	18	11	9	13	9
Mean ABG (dB)	21	22	19	21	10	21
Mean gain in AC (dB)	18	19	16	20	22	20
SNHL % (n)	0	NA	9 (2)	6 (1)	0	0
ABG ≤20 dB % (n)	44.4 (8)	56.1 (23)	65 (15)	61 (11)	75 (12)	57.9 (11)

*Note*. Class 3 anomaly results—current study compared to the literature. All results (current and in the literature) are based on audiometry PTAs using 0.5, 1, 2, and 3 or 4 kHz.

### Strengths and Limitations of this Study

In this study we analyzed the results of reconstructive surgery on hearing for all surgically treated CMEA patients in our medical center. The detailed description and analysis of included cases provides knowledge on the expected outcome of reconstructive surgery of the ossicular chain in these patients. Though, several limitations needs to be considered. First, we included a cohort of children and adults who were surgically treated for CMEAs. Thereby, outcomes could differ to previously published hearing outcomes in cohorts only including children,^20,22,29,31,32^ or excluding those patients with mixed hearing loss, inner ear anomalies, otologic comorbidities and a history of recurrent otitis media.^5,6,10,13-16,20,22,33^ Second, long term hearing outcome of 10 months or longer after surgery was only available for half the cohort (9 out of 18 patients).

### Decision Making and Surgical Indications in CMEAs

In this retrospective chart review, we demonstrated that surgery is a clinically relevant treatment option for CMEAs to reach AC thresholds below 30 dB to achieve functional hearing levels. Furthermore, even if AC PTAs remain >30 dB, improved hearing can be of importance to the patient by facilitating additional hearing amplification by hearing aids.^34^ Exclusive non-surgical hearing improvement might be considered in unilateral patients in which postoperative hearing gain is less likely, for example, patients with extensive otologic histories, with class 4 anomalies, with specific syndromal diagnoses, and in patients with inner ear deformities.^4^ Also, the importance of preoperative radiological imaging and careful inspection of the ossicular chain during ET for additional fixations/malformations needs to be mentioned as these factors could hinder successful outcome.

## Conclusions

Surgery proved to be an effective treatment option to restore hearing for CMEAs. Surgical goals of obtained gain in AC thresholds and reached serviceable hearing levels were met by most patients without any iatrogenic sensorineural hearing losses or other inner ear problems.
